# Facebook addiction and sleep problems in peruvian university students after the COVID-19 pandemic

**DOI:** 10.1016/j.heliyon.2024.e24383

**Published:** 2024-01-09

**Authors:** Joel Figueroa-Quiñones, Willy Valle-Salvatierra, Condor Heredia Nelly Teresa

**Affiliations:** a Universidad Señor de Sipán, Chiclayo, Peru; b Escuela Profesional de Psicología, Universidad Católica Los Ángeles de Chimbote, Chimbote, Peru; c Escuela Profesional de Enfermería, Universidad Cesar Vallejo, Piura, Peru

**Keywords:** Facebook addiction, COVID-19, Sleep problems, College students

## Abstract

**Background:**

During the COVID-19 pandemic, studies have reported an increase in sleep problems and problematic use of social media platforms such as Facebook among university students. This study assessed Facebook addiction and sleep problems among Peruvian university students following the COVID-19 pandemic, as well as the factors associated with these issues.

**Methods:**

A cross-sectional study was conducted with a sample of 352 participants from different regions of Peru. The Jenkins Sleep Scale (JSS-4) and the Bergen Facebook Addiction Scale (BFAS) were used to assess sleep problems and Facebook addiction, respectively. Prevalence ratios (PR) were calculated using a simple Poisson regression with robust variance**.**

**Results:**

The study found that 16.2 % of the participants were addicted to Facebook and 12.5 % reported sleep problems. The results also showed that older age (PR: 0.99; 95 % CI: 0.98–0.99) and physical activity (PR: 0.81; 95 % CI: 0.70–0.94) were associated with a lower likelihood of having sleep problems, while being physically active (PR: 0.55; 95 % CI: 0.33 to 0.90) was associated with a lower probability of having Facebook addiction problems.

**Conclusions:**

The Peruvian university students who participated in this study reported sleep problems in one-eighth of the sample, and one in six university students reported Facebook addiction problems. The frequency of presenting Facebook addiction and sleep problems was lower in those with older age and engaging in physical activity.

## 1 Introduction

The World Health Organization (WHO) declared the COVID-19 pandemic globally, causing tremendous alarm and concern. Different measures were implemented to reduce the spread of this disease [1]. The lethality of COVID-19 varies by age, gender, and the overall health of the infected person. Generally, older people and those with pre-existing medical conditions such as cardiovascular disease, diabetes, or cancer are at higher risk of developing severe complications and dying from the disease [2]. However, even young and healthy people can become seriously ill and die from COVID-19. Additionally, the lethality of the disease can vary depending on the location and timing of the outbreak, as factors such as access to healthcare and the capacity of healthcare systems to treat affected patients affect the mortality rate. In general, it is estimated that the fatality rate of COVID-19 may be higher in certain population groups [3].

The pandemic has affected all regions of the world, but some have been more severely affected than others. Initially, the pandemic had a devastating impact in China, where the outbreak originated. However, in the following months, COVID-19 spread to other parts of the world, especially Europe, which became the epicenter of the pandemic in the spring of 2020 [4]. Later on, the pandemic spread to North and South America, where the United States, Brazil, and Mexico became the most affected countries in terms of the number of cases and deaths. The region also experienced significant outbreaks in countries such as Peru, Colombia, Argentina, and Chile. Overall, the regions most affected by COVID-19 were those with high population densities and health systems that are less prepared to handle a pandemic [5].

However, few years after its declaration, evidence has emerged of its physical and emotional impact on various populations, including young people and adults, who were more exposed to work, academic, and family responsibilities that they had to cope with during this time [6,7]. For instance, the COVID-19 pandemic has had a profound impact on the education of millions of young people around the world. The sudden shift to online learning has posed numerous challenges, including limited access to technology, reduced social interaction, and concerns about the quality of education [8,9]. Moreover, young people have been disproportionately affected by the pandemic in terms of employment. Many jobs typically held by young people, such as those in customer service, restaurants, and retail, have been disrupted or eliminated, leading to heightened levels of stress, anxiety, and uncertainty [10]. Furthermore, restrictions limited opportunities for young people to socialize and engage in face-to-face social activities, resulting in feelings of isolation, loneliness, and a lack of opportunities to develop social skills [11].

During the COVID-19 pandemic, studies have reported an increase in sleep problems and affectation among university students [12]. For instance, a review study found a prevalence of insomnia in 27.3 % of Chinese university students [13]. These problems are known to arise due to worry, distress, family issues, work conditions, and other factors associated with sleep disruption [14]. Studies suggest that sleep deprivation is linked to poor academic performance, as the student may have difficulties with attention and concentration [15]. Moreover, diseases such as diabetes, hypertension, cardiovascular, and cerebrovascular problems are associated with decreased sleep [16]. These issues may have been exacerbated during the lockdown, as a significant portion of the population altered their sleep habits and patterns [17].

During the COVID-19 lockdown, prolonged use of online activities has been reported, and a trend towards problematic use has emerged [18]. For example, the use of social media has become one of the major platforms in which online users invest their time [19]. In fact, a review reports that during the pandemic, the prevalence of social media addiction was 15.1 %, mainly among young people [20]. As of 2022, Facebook maintains its popularity with over 2.963 billion monthly active users [21]. Therefore, various studies have shown that excessive use of social media has been associated with emotional and academic problems [22,23].

Facebook addiction involves compulsive behavior and an excessive dependence on this social media platform [24]. Some studies have revealed that addiction to social media platforms like Facebook is related to sleep problems and discomfort [25]. In fact, a study concluded that people who tend to be addicted to Facebook are at a higher risk of experiencing sleep disorders [26,27]. Some factors such as physical inactivity, deficit of skills, depression or anxiety, could increase the risk of presenting these problems [28,29]. Therefore, physical health and psychological well-being of young people may have been more affected by the events experienced during the COVID-19 pandemic [30].

In Peru, the pandemic impacted public health severely, with a high number of cases and deaths. The Peruvian health system was overwhelmed by the number of patients needing medical attention, which led to a lack of beds, oxygen, and other resources needed to treat the sick [30]. The Peruvian education system was affected, with the closure of schools and universities and the transition to distance education. This generated inequalities in access to education and the technology necessary for online learning [31]. In addition, the pandemic had a negative impact on Peruvian economy, especially in the tourism sector and small and medium-sized businesses. The lockdown and restrictions imposed to contain the spread of the virus led to job losses and decreased production and commerce [32].

In Peru, according to the Institute of Public Opinion Surveys [33], 80 % of the urban population are users of social media, with Facebook being the second most frequently used platform. Some studies in the general population of Peru have reported a prevalence of social media addiction at 23.6 % [34], and Facebook addiction was found in 8.6 % of university students [35]. On the other hand, sleep problems during the pandemic have been reported in up to 60.1 % of university students [36]. Only one pre-pandemic study reported the association between Facebook dependence and poor sleep quality in university students from Lima [35]. Currently, there is evidence on the social, economic, and physical effects of the COVID-19 pandemic on university students [37,38]. However, to date, no study has evaluated both variables and their associated factors following the COVID-19 pandemic, despite the fact that prolonged exposure to social media sites, especially before bedtime, can disrupt sleep patterns and make it difficult to get adequate rest. This may have a negative impact on daily responsibilities, work capacity and learning in the university population [39,40]. Therefore, there is a need to address the factors associated with Facebook addiction and sleep disorders in college students. This will provide a solid foundation for the design of effective policies and intervention strategies in this population.

Therefore, this study assessed the factors associated with Facebook addiction and sleep problems in Peruvian university students following the COVID-19 pandemic.

## 2 Methods

### 2.1 Study design

A cross-sectional analytical study was conducted to assess the factors associated with Facebook addiction and sleep problems among Peruvian university students after the COVID-19 pandemic.

## 3 Participants

The population consisted of university students from the coast (Ancash, Arequipa, Callao, Ica, Lambayeque, La Libertad, Lima, Moquegua, Piura, Tumbes, and Tacna), highlands (Apurímac, Ayacucho, Cajamarca, and Puno), and jungle (Amazonas, Ucayali, and Loreto) regions of a private university in Peru. Participants were selected through intentional non-probabilistic sampling, and included those who: 1) were over 18 years old, 2) agreed to participate in the study, 3) completed all the questions. Participants with incomplete responses were excluded. A statistical power analysis was applied with the number of predictors necessary to determine the recommended sample size. The G*Power version 3.1.9.7 program was used, with a significance level (α) of 0.05, specifying an effect size of 0.08, and a power of 0.9. The "N" required for the study was 236 subjects.

Variables.

The Jenkins Sleep Scale-4 (JSS-4) was used to evaluate sleep problems. This instrument is unidimensional and consists of 4 items and a Likert-type scoring from 0 (does not occur to me) to 5 (occurs to me from 22 to 31 days). The score is obtained directly by adding up the obtained raw scores. In this sense, a score of 11 is a cut-off point: a score <12 is defined as little sleep disturbance, and a score >11 is understood as high frequency of sleep disturbances [41]. Likewise, the presence of sleep difficulties was considered if there was at least one affirmative response (>15 nights in the previous 4 weeks) in any item [40]. For this study, the adapted version for Peru with adequate psychometric properties (α = 0.82, ω = 0.83) was used [42].

The Bergen Facebook Addiction Scale (BFAS) was originally developed by Andreassen et al., in 2012 to evaluate addictive problems [43]. In this study, the short unidimensional version of 6 items with 5 response options ranging from 5 (very often) to 1 (very rarely) was used. Problematic use was dichotomized with scores >18. The unidimensional version has shown adequate psychometric properties in the Peruvian population with a α = 0.84 [44].

The covariates included in the study were age (years), place of residence (coast, highlands, and jungle), gender (male or female), belonging to a religion (yes or no), working status (yes or no), having a partner (yes or no), and engaging in physical activity (yes or no).

## 4 Procedures

We developed an online questionnaire hosted on Google Forms, as university classes were being held virtually. The questionnaire included a consent form for participants, the study objectives, and the scales. Data collection was conducted during November and December 2022. We requested the cooperation of the university faculty to share the questionnaire link via WhatsApp study groups, and we also sent emails to request participation.

### 4.1 Data analysis

The sociodemographic characteristics, Facebook addiction levels, and sleep problems were processed and analyzed using descriptive statistics techniques. The results were presented and summarized in texts and tables of relative and absolute frequencies for categorical variables and measures of central tendency and dispersion for numerical variables.

To assess factors associated with Facebook addiction and sleep problems, prevalence ratios (PR) and 95 % confidence intervals (CI) were calculated using a simple Poisson regression with robust variance. Only variables with a p-value <.20 in the crude model were included in the adjusted model. The statistical analysis was performed using the R studio software platform.

### 4.2 Ethics

This study was reviewed and approved by the Research Ethics Committee of Universidad Católica Los Ángeles de Chimbote under No. 112-2022-CIEI-VI-ULADECH-CATOLICA. In addition, informed consent was obtained from participants and their data were protected through confidentiality and secure responses stored in an Excel file with password access only granted to the principal investigator of the study.

## 5 Results

A total of 357 participants were surveyed, 5 of whom did not complete the questionnaire, resulting in a final sample of 352 participants (response rate of 98.6 %). The university students had a median age of 27 (interquartile range: 22–34 years). The distribution by residence showed a higher prevalence of participants from the coast with a total of 239 (67.9 %), while the highlands and jungle region had 81 (23.0 %) and 32 (9.1 %) respectively. Female participants had a greater representation with 258 (73.3 %), 226 (64.2 %) reported being religious, 240 (68.2 %) had a job, 197 (56 %) had a partner, and 178 (50.6 %) engaged in physical activity. In addition, 44 (12.5 %) reported having sleep problems and 57 (16.2 %) reported problematic levels of Facebook use (Table 1).

**Table 1:** Characteristics of the participants.

Factors	N = 352
Age^a^	27 [[22], [23], [24], [25], [26], [27], [28], [29], [30], [31], [32], [33], [34]]
Residence	
Coast	239 (67.9)
Highlands	81 (23.0)
Jungle	32 (9.1)
Sex	
Female	258 (73.3)
Male	94 (26.7)
Religious	
Yes	226 (64.2)
Not	126 (35.8)
Working	
Yes	240 (68.2)
Not	112 (31.8)
With couple	
Yes	197 (56.0)
Not	155 (44.0)
Physical Activity	
Yes	178 (50.6)
Not	174 (49.4)
Sleeping problems (JSS-4)	
No problems	308 (87.5)
With problems	44 (12.5)
Facebook addiction (BFAS)	
No problems	295 (83.8)
Problematic use	57 (16.2)

a Median and Interquartile range.

Table 2 shows the factors associated with sleep problems and Facebook addiction. In the crude model, we obtained that Facebook addiction was lower among residents of the jungle region of Peru (PR: 0.20; 95 % CI: 0.02–0.84) compared to residents of the coast. However, after the adjusted model this factor was not held. In addition, students who reported physical activity had a lower prevalence of problematic Facebook use (PR: 0.55; 95 % CI: 0.33–0.90). On the other hand, it was found in the crude and adjusted model that both age (PR: 0.99; 95 % CI: 0.98–0.99) and physical activity (PR: 0.81; 95 % CI: 0.70–0.94) were factors statistically associated with sleep problems.

**Table 2:** Factors associated with Facebook addiction and sleep problems in Peruvian university students following the COVID-19 pandemic.

Factors	Facebook addiction				Sleeping problems			
	RP crude (IC 95 %)	p value	RP Adjusted (IC 95 %)	p value	RP crude (IC 95 %)	p value	RP Adjusted (IC 95 %)	p value
**Age**	0.97 (0.94–1.00)	0.07	0.98 (0.94–1.00)	0.16	0.99 (0.98–1.00)	0.00	0.99 (0.98–0.99)	0.00
**Residence**								
Coast	Ref.				Ref.			
Highlands	1.52 (0.89–2.49)	0.11	1.48 (0.87–2.46)	0.13	0.95 (0.79–1.14)	0.60	–	–
Jungle	0.20 (0.02–0.84)	0.08	0.23 (0.02–0.95)	0.11	0.97 (0.74–1.26)	0.85	–	–
**Sex**								
Male	0.81 (0.44–1.40)	0.47	–	–	0.99 (0.83–1.67)	0.90	–	–
Female	Ref.				Ref.			
**Religion**								
Not	Ref.				Ref.			
Yes	0.66 (0.41–1.08)	0.09	0.71 (0.44–1.15)	0.15	1.05 (0.90–1.23)	0.51	–	–
**Working**								
Not	Ref.				Ref.			
Yes	1.20 (0.72–2.08)	0.50	–	–	0.95 (0.81–1.12)	0.54	–	–
**With couple**								
Not	Ref.				Ref.			
Yes	0.76 (0.47–1.23)	0.25	–	–	1.02 (0.88–1.18)	0.81	–	–
**Physical activity**								
Not	Ref.				Ref.			
Yes	0.57 (0.34–0.93)	0.02	0.55 (0.33–0.90)	0.02	0.81 (0.70–0.94)	0.00	0.81 (0.70–0.94)	0.00

IC95 %: confidence interval; RP: Prevalence ratio, Ref: reference category.

## 6 Discussion

The aim of this study was to determine the factors associated with Facebook addiction and sleep problems in university students in the coast, highland and jungle regions of Peru after the COVID-19 pandemic. In addition, the study presented the reported prevalences of both problems. According to the study, a significant proportion of participants were addicted to Facebook (16.2 %) and experienced sleep problems (12.5 %). Further analysis showed that older age (PR: 0.99; 95 % CI: 0.98–0.99) and physical activity (PR: 0.81; 95 % CI: 0.70–0.94) were associated with a lower likelihood of having sleep problems, while being physically active (PR: 0.55; 95 % CI: 0.33–0.90) and living in the Peruvian jungle (PR: 0.23; 95 % CI: 0.02–0.95) were associated with a lower probability of having Facebook addiction problems, although the latter factor in the adjusted model was not held.

University students reported Facebook addiction problems in 57 (16.2 %) cases. These findings are consistent with a study conducted on Bangladeshi university students after the COVID-19 pandemic, which reported a prevalence of Facebook addiction at 29.4 % [45]. Another study on Trinidadian university students reported that 8.5 % presented a high risk of Facebook addiction [46]. In Brazil, a study found that 32 % of the population qualified as addicted to Facebook [47], and in Colombia, a study found that 29.3 % of men and 22.4 % of women reported problematic use of Facebook [48]. These findings are likely due to several factors. For example, despite the lifting of isolation measures, academic activities have remained virtual following the COVID-19 pandemic, and Facebook is the most widely used social network by young people for social interaction [49]. In addition, people can spend more time on the platform due to the accessibility of technology and the rise in the use of mobile devices with the ease of accessing Facebook anytime and anywhere through mobile phones. Another factor that contributes to Facebook addiction is the design of the platform [50]. Social media, including Facebook, are designed to be highly engaging and addictive. Constant notifications, positive feedback through "likes" and comments, and the feeling of connection with other users can increase dopamine in the brain, which can lead to addictive behavior [51]. In addition, the nature of social media can create a sense of FOMO (fear of missing out) among users. Seeing the posts of friends and acquaintances, people may feel compelled to keep checking Facebook to make sure they do not miss any important updates or events [52].

The JSS-4 indicated that 44 (12.5 %) had sleep problems. Similarly, studies on the Egyptian population and Polish university students reported sleep problems in 5.4 % and 21.6 %, respectively, during the COVID-19 pandemic [53,54]. In Colombia, a study found a high prevalence of 36.2 % of sleep problems in its population; however, this is likely due to the fact that the participants were healthcare workers and this group was severely affected by the pandemic [55]. Likewise, another study with Argentinean university students reported prevalences of up to 23 % of moderate insomnia problems in academics (56). On the other hand, sleep problems have multifactorial causes [56]. For example, the academic workload and pressure to meet academic expectations can create high levels of stress, which can affect sleep [57]. In addition, many university students have an unhealthy lifestyle, including an unbalanced diet, lack of exercise and sedentary activities, excessive use of technology and social media, and drug or alcohol consumption [58]. Similarly, irregular schedules, travel, part-time jobs, and social activities can disrupt sleep patterns and contribute to insomnia [59]. Finally, comorbid mental health problems such as anxiety, depression, and other mental health disorders can interfere with sleep and contribute to insomnia [36].

It was found that for every additional year, the prevalence of reporting sleep problems was 1 % lower (PR: 0.99; 95 % CI: 0.98–0.99). This finding coincides with a study in medical university students in Poland, which reported that those with a higher age were less likely to report insomnia symptoms [60]. Another study in Ethiopia indicates that younger students had higher scores on insomnia problems [61]. It is likely that these findings are due to younger students using nighttime hours for web browsing, social media, entertainment platforms, as well as academic activities or online gaming, which disrupts sleep habits [[62], [63], [64], [65]]. Additionally, there are other factors that could precipitate sleep problems in this population, such as pressure or stress generated by maintaining adequate grades or economic and/or family difficulties [[66], [67], [68]].

Furthermore, university students who engaged in physical activity reported 19 % fewer sleep problems compared to sedentary students (PR: 0.81; 95 % CI: 0.70–0.94). This result is consistent with a study of Polish university students, in which a lower prevalence of insomnia symptoms was found in those who reported higher levels of physical activity [52]. Similarly, a study of Basque schoolchildren reported that those who engaged in some form of physical activity or sports had better sleep efficiency [69]. A review suggests that physical activity reduces stress, facilitating sleep initiation [70]. Additionally, there is evidence that physical activity reduces mental health problems, which are associated with sleep problems. Therefore, reducing these symptoms could improve sleep quality [71,72]. Likewise, a review shows that after physical exercise, body temperature decreases, promoting sleep [73].

The frequency of Facebook addiction was lower in university students residing in the Peruvian jungle compared to those on the coast (PR: 0.23; 95 % CI: 0.02–0.95). This result could be explained by the fact that the economy of jungle university students was more affected during the COVID-19 pandemic, leading them to take on work responsibilities to cover their educational and family expenses, resulting in less time available for social media use [74]. Additionally, access and connectivity in the jungle region of Peru are limited, which could reduce the interest in staying active on social media and the internet [75]. Furthermore, we cannot rule out the underestimation of the real prevalence, given that the sample of participants from the jungle was smaller [76].

On the other hand, students who engaged in physical activity reported a lower prevalence of problematic use of Facebook (PR: 0.55; 95 % CI: 0.33–0.90). A study with medical students in Turkey reported that engaging in physical activity at least twice a week was a preventive factor for internet addiction [77]. Furthermore, other studies have reported that increasing physical activity decreases Facebook addiction [22]. This finding could be explained by the fact that engaging in physical activity contributes to social interaction, as these activities are often done in groups, thus reducing the need to browse social media [79]. Additionally, it is likely that engaging in physical activity reduces the time available for other activities such as social media use [80].

It is important to point out some strengths in this study, this is the first study conducted after the COVID-19 pandemic that investigates factors associated with Facebook addiction and sleep problems in a population of university students in Peru. Therefore, public health campaigns and interventions aimed at increasing the quality of sleep and proper use of Facebook can be generated. In addition, information was collected from an optimal sample of students residing in the coast, highlands and jungle regions of Peru using standardized and validated questionnaires for both measures, thus our findings are representative and objective. However, it is important to keep in mind some limitations. For example, we did not include questions on mental health, medication use among students, or substance use such as alcohol and tobacco. Consequently, sleep problems may have been influenced by these and other covariates that primarily affect young populations. Therefore, future studies should delve deeper into medical history, lifestyle factors, mental health treatment, and sleep problems. Furthermore, by using a non-probability sampling method, the generalizability of the results of this study is limited. And by using a cross-sectional design in the study, the results should be interpreted with caution.

Based on our findings on sleep problems and Facebook addiction among Peruvian university students that occurred after the COVID-19 pandemic, a current overview of these problems is obtained from which future studies could evaluate interventions and immediate actions for the most vulnerable groups affected. For example, it is important that universities, through their tutoring and student well-being departments, provide student-focused monitoring and counseling to educate and prevent sleep problems and Facebook addiction. Furthermore, the findings of this study suggest that interventions aimed at reducing sleep disturbances or Facebook addiction should consider physical activity as a mediator. Also, subsequent studies could delve deeper into the topic by exploring other social networks such as Tiktok or Instagram.

In the same way, professional psychology associations nationwide, together with professionals who work in communities or academic institutions, should offer workshops and talks aimed at parents, since this type of behavior could be prevented from childhood. Moreover, it is suggested that professionals and parents take into consideration the following topics: 1) Supervision and control of the use of Facebook in order to establish schedules and improve the habits of use of this social network. 2) Promotion of other types of leisure activities such as reading and even face-to-face socialization, and physical exercise.

## 7 Conclusions

In conclusion, one in six Peruvian university students reported Facebook addiction problems, and one eighth reported sleep problems. When evaluating the factors associated with sleep problems and Facebook addiction, it was found that the frequency of these problems was lower in those who were physically active and older.

## Data availability statement

The data will be available upon request addressed to the main author.

## Additional information

No additional information is available for this paper.

## CRediT authorship contribution statement

**Joel Figueroa-Quiñones:** Writing – review & editing, Writing – original draft, Visualization, Validation, Supervision, Software, Resources, Project administration, Methodology, Investigation, Funding acquisition, Formal analysis, Data curation, Conceptualization. **Willy Valle-Salvatierra:** Writing – review & editing, Writing – original draft, Project administration, Methodology, Investigation, Data curation, Conceptualization. **Condor Heredia Nelly Teresa:** Writing – original draft, Supervision, Funding acquisition, Data curation.

## Declaration of competing interest

The authors declare that they have no known competing financial interests or personal relationships that could have appeared to influence the work reported in this paper.
